# Supplementary material for: Hammerstein–Wiener Motion Artifact Correction for Functional Near-Infrared Spectroscopy: A Novel Inertial Measurement Unit-Based Technique
Source: Sensors (Basel). 2024 May 16;24(10):3173. doi: 10.3390/s24103173 (PMC11125330; doi:10.3390/s24103173)
Supplement: Supplementary file 1 [file sensors-24-03173-s001.zip › sensors-2907267-supplementary.pdf]

Supplementary Materials

Arduino codes:

```
#include<Wire.h>

const int MPU2=0x69,MPU1=0x68;

int16_t AcX1,AcY1,AcZ1,Tmp1,GyX1,GyY1,GyZ1;

int16_t AcX2,AcY2,AcZ2,Tmp2,GyX2,GyY2,GyZ2;

//------------------------------------------------\setup loop\------------------------------------------------------------

 void setup(){

   Wire.begin();

   Wire.beginTransmission(MPU1);

   Wire.write(0x6B);// PWR_MGMT_1 register

   Wire.write(0); // set to zero (wakes up the MPU-6050)

   Wire.endTransmission(true);

   Wire.beginTransmission(MPU2);

   Wire.write(0x6B);// PWR_MGMT_1 register

   Wire.write(0); // set to zero (wakes up the MPU-6050)

   Wire.endTransmission(true);

   Serial.begin(9600);

  }

//--------------------------------------------------\void loop\----------------------------------------------------------
--

 void loop(){

   //get values for first mpu having address of 0x68

   GetMpuValue1(MPU1);
```

```
      Serial.print("");
      //get values for second mpu having address of 0x69
      GetMpuValue2(MPU2);
      Serial.println("");
     }
   //-------------------------------------------\user defined functions\---------------------------------------------
--

   void GetMpuValue1(const int MPU){
      Wire.beginTransmission(MPU);
      Wire.write(0x3B); // starting with register 0x3B (ACCEL_XOUT_H)
      Wire.endTransmission(false);
      Wire.requestFrom(MPU, 14, true); // request a total of 14 registers
      AcX1=Wire.read()<<8| Wire.read(); // 0x3B (ACCEL_XOUT_H) & 0x3C (ACCEL_XOUT_L)
      AcY1=Wire.read()<<8|  Wire.read(); // 0x3D (ACCEL_YOUT_H) & 0x3E (ACCEL_YOUT_L)
      AcZ1=Wire.read()<<8| Wire.read(); // 0x3F (ACCEL_ZOUT_H) & 0x40 (ACCEL_ZOUT_L)
      GyX1=Wire.read()<<8| Wire.read(); // 0x43 (GYRO_XOUT_H) & 0x44 (GYRO_XOUT_L)
      GyY1=Wire.read()<<8| Wire.read(); // 0x45 (GYRO_YOUT_H) & 0x46 (GYRO_YOUT_L)
      GyZ1=Wire.read()<<8| Wire.read(); // 0x47 (GYRO_ZOUT_H) & 0x48 (GYRO_ZOUT_L)
     }
   void GetMpuValue2(const int MPU){
      Wire.beginTransmission(MPU);
      Wire.write(0x3B); // starting with register 0x3B (ACCEL_XOUT_H)
      Wire.endTransmission(false);
      Wire.requestFrom(MPU, 14, true); // request a total of 14 registers
      AcX2=Wire.read()<<8| Wire.read(); // 0x3B (ACCEL_XOUT_H) & 0x3C (ACCEL_XOUT_L)
      AcY2=Wire.read()<<8|  Wire.read(); // 0x3D (ACCEL_YOUT_H) & 0x3E (ACCEL_YOUT_L)
      AcZ2=Wire.read()<<8| Wire.read(); // 0x3F (ACCEL_ZOUT_H) & 0x40 (ACCEL_ZOUT_L)
      GyX2=Wire.read()<<8| Wire.read(); // 0x43 (GYRO_XOUT_H) & 0x44 (GYRO_XOUT_L)
      GyY2=Wire.read()<<8| Wire.read(); // 0x45 (GYRO_YOUT_H) & 0x46 (GYRO_YOUT_L)
      GyZ2=Wire.read()<<8| Wire.read(); // 0x47 (GYRO_ZOUT_H) & 0x48 (GYRO_ZOUT_L)
```

```
//MPU-1
Serial.print("  ");Serial.print(AcX1);
Serial.print(" ,");Serial.print("  ");Serial.print(AcY1),
Serial.print(" ,");Serial.print("  ");Serial.print(AcZ1),
Serial.print(" ,");Serial.print("  ");Serial.print(GyX1),
Serial.print(" ,");Serial.print("  ");Serial.print(GyY1),
Serial.print(" ,");Serial.print("  ");Serial.print(GyZ1),
//MPU-2
Serial.print(" ,");Serial.print("  ");Serial.print(AcX2),
Serial.print(" ,");Serial.print("  ");Serial.print(AcY2),
Serial.print(" ,");Serial.print("  ");Serial.print(AcZ2),
Serial.print(" ,");Serial.print("  ");Serial.print(GyX2),
Serial.print(" ,");Serial.print("  ");Serial.print(GyY2),
Serial.print(" ,");Serial.print("  ");Serial.print(GyZ2),
delay(1);
}
```

Table S1: MA correction processing durations

The processing durations for 41.5 minutes of data were recorded in 8 channels (8 HbO and 8 HbR) sampled at 10 Hz. The analysis was run with HOMER3 on a PC with a Windows 10 operating system and an intel® core™ i5-7500 @3.4 GHz processor.

Table S1. The processing time for each tested technique.

Methods	Processing time (seconds)
Uncorrected	-
RLOESS	700 ±6
WCBSI	65 ±0.2
Wavelet	62 ±0.2
HWM (head-IMU or probe-IMU)	26 ±6.2
splineSG	21 ±0.3
PCA	17 ±0.5
Spline	17 ±0.4
tPCA	16 ±0.5
CBSI	16 ±0.5

Table S2: Participants.

Table S2. The participant's age, gender, and hair colour

participant	Age	gender	Hair colour
sub-01	34	Male	dark brown
sub-02	34	Female	black
sub-03	26	Female	medium brown
sub-04	33	Female	black
sub-05	29	Female	light brown
sub-06	26	Female	brown
sub-07	29	Male	very dark brown
sub-08	34	Male	black
sub-09	29	Female	brown
sub-10	26	Female	black
sub-11	32	Female	black
sub-12	34	Male	brown
sub-13	29	Female	red
sub-14	24	Male	light brown
sub-15	29	Female	brown
sub-16	24	Female	dark brown
sub-17	24	Female	black
